# A New Shear-Stress-Based Point-of-Care Technology for Evaluation of the Hemostatic Pattern in Whole Blood

**DOI:** 10.3390/bios14110518

**Published:** 2024-10-22

**Authors:** Alessandro Foladore, Simone Lattanzio, Elisabetta Lombardi, Cristina Durante, Ekaterina Baryshnikova, Martina Anguissola, Lidia Rota, Marco Ranucci, Mario Mazzucato

**Affiliations:** 1 Sedicidodici s.r.l., 33170 Pordenone, Italy; afoladore@1612medical.com (A.F.); slattanzio@1612medical.com (S.L.); lidiarotavender@gmail.com (L.R.); 2 Stem Cell Unit, Department of Research and Advanced Cancer Diagnostic, Centro di Riferimento Oncologico di Aviano (CRO), IRCCS, 33081 Aviano, Italy; elombardi@cro.it (E.L.); cdurante@cro.it (C.D.); moreno.mazzucato@libero.it (M.M.); 3 Department of Cardiothoracic and Vascular Anesthesia and Intensive Care, Istituto di Ricovero e Cura a Carattere Scientifico, Policlinico San Donato, 20097 San Donato Milanese, Italy; ekaterina.baryshnikova@grupposandonato.it (E.B.); martina.anguissola@grupposandonato.it (M.A.)

**Keywords:** coagulation, point of care, image analysis, shear rate, platelets, fibrin

## Abstract

The currently available point-of-care hemostasis tests are burdened by criticisms concerning the use of different activators and inhibitors and the lack of dynamic flow. These operating conditions may constitute an impediment to the determination of the patient’s hemostatic condition. Hence, the diffusion of these tests in clinical practice is still limited to specific scenarios. In this work, we present a new method for analyzing the patient’s global hemostasis based on the visualization of the main components of the coagulation process and its computerized quantitative image analysis. The automated “Smart Clot” point-of-care system presents a micro-fluidic chamber in which whole blood flows, without the addition of any activator or inhibitor. In this micro-channel, platelet adhesion, activation and aggregation to the type I collagen-coated surface take place (primary hemostasis), leading to the production of endogenous thrombin on the surface of platelet aggregates and the consequent fibrin mesh and thrombus formation (secondary hemostasis). These observations are verified by inhibiting primary hemostasis with the antiplatelet drugs Indomethacin (−70% on platelet aggregation, −60% on fibrin(ogen) formation) and Tirofiban (complete inhibition of platelet aggregation and fibrin(ogen) formation) and secondary hemostasis with the antithrombin drugs Heparin (−70% on platelet aggregation, −80% on fibrin(ogen) formation) and Lepirudin (−80% on platelet aggregation, −90% on fibrin(ogen) formation). Smart Clot, through a single test, provides quantitative results concerning platelet aggregation and fibrin formation and is suitable for undergoing comparative studies with other coagulation point-of-care devices.

## 1. Introduction

Point-of-care (POC) tests for the assessment of the hemostatic and coagulation process are currently used in specific clinical scenarios. They are mainly applied to bleeding patients in critical areas (cardiac surgery, liver transplant, trauma, severe sepsis, post-partum hemorrhage, and others). Their application to identify the mechanism(s) underlying bleeding and to suggest the therapeutic approach is sustained by the existing guidelines in the field of cardiac surgery [1,2], major surgery [3], trauma [4], and post-partum hemorrhage [5].

There are numerous coagulation POC devices available on the market. The majority are viscoelastic tests, which, using different kinds of activators (kaolin, tissue factor, ellagic acid, reptilase), inhibitors (Heparin, abciximab, cytochalasin D) and enzymes (heparinase), provide an analysis of the viscoelastic properties of the clot, generally expressed in terms of the reaction time (the time from the start of the test to the first fibrin network formation), clot strength (mm or hPa), fibrin contribution to the clot strength (skipping platelet contribution), platelet contribution to the clot strength (difference between the last two), and clot lysis (kinetics of the decrease in clot strength from the maximum).

Additionally, there are devices dedicated to platelet function analysis, including multi-electrode aggregometry and others.

The common characteristics of the existing coagulation POC tests are (i) the use of whole blood; (ii) the use of one or more activators; (iii) the ability to provide a response within 10–20 min or even less; and (iv) for viscoelastic test, the expression of the results based on the physical properties of the clot.

Several new-generation microfluidic-based devices have been proposed for coagulation studies [6,7,8,9]. These devices constitute advantageous research platforms; however, they yield different characteristics to the Smart Clot. These differences, in terms of the possible advantages and limitations, will be addressed in the Discussion.

Hemostasis is a complex system that adapts to the various hemodynamic conditions that occur in the circulatory system. To stop bleeding, in the event of vascular damage, several steps are involved to ensure that blood loss is minimized without causing thrombosis and while maintaining normal blood flow [10]. The traditional methods ignore the presence of underlying mechanical forces that are crucial to the normal physiological response [11,12].

Blood flows in the vasculature, like any other fluid, generating mechanical shear stress on the walls containing the fluid [13]. This wall shear stress has been shown to affect not only cells flowing along the vascular wall but also circulating blood proteins [14,15]. Evaluation of shear stress can effectively be used in experiments through the use of microfluidic technology.

Novel systems, as close as possible to human in vivo conditions, integrating all the important components of thrombus formation, such as blood cells, cell fragments, coagulation factors and physiological generation of endogenous activators, extracellular matrix and dynamic flow conditions, are needed to improve our understanding of coagulation pathophysiology and the role of the individual components in the global process. No device is presently providing a visual representation of the clot and its elements. The present report presents a new-generation coagulation POC device based on the use of a micro-channel flow chamber and on the computerized image analysis of the clot’s components, integrated with a numerical output of the reaction time, and of the fibrin(ogen)’s and platelets’ contribution to the global clot composition. Smart Clot is a novel, fully automated device able to evaluate the global hemostatic condition of each patient, simultaneously evaluating both platelets’ (primary hemostasis) and fibrin’s (secondary hemostasis) contribution to the coagulation process without the addition of exogenous agonists, in accordance with the recommendations of the “Biorheology Subcommittee of the SCC of the ISTH”, in order to standardize flow-chamber-based assays to measure thrombus formation in vitro [16].

## 2. Materials and Methods

### 2.1. Device Description

**Figure 1:**
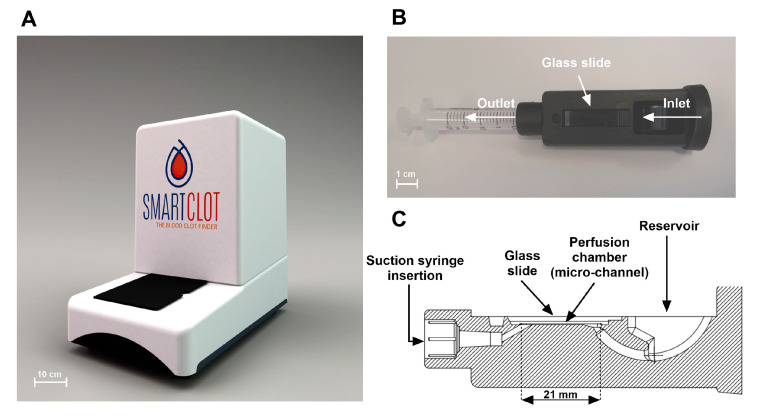
The Smart Clot technology: (**A**) the Smart Clot instrument; (**B**) the Smart Clot cartridge; and (**C**) vertical section of the Smart Clot cartridge (21 mm: length of the micro-channel).

The Smart Clot system is composed by an instrument (Figure 1A) equipped with a real-time computerized video-imaging analysis system, Smart Clot software (version 30.0.7) and a disposable single-use kit (Smart Clot Automation Kit–Platelets and Fibrin Detection). The Smart Clot instrument and software were built by A.S.T. Biomedical (Albino, Italy), following Sedicidodici’s studies, technical design and specifications. The device measures 575 × 372 × 657mm (H × W × D).

The hardware of the Smart Clot system is composed of an embedded processor, an optical system equipped with a B/W CMOS camera (C13752-50U, Hamamatsu Photonics, Hamamatsu, Japan), an automated pipette for liquid handling, a reagent and sample support module, a movable, heated cartridge support module, a syringe management module (constituted by a linear suction pump), a waste container module, a set of automated sensors for the purpose of checking the cartridge, reagent, sample and waste container loading, and a set of automated sensors for the purpose of mechanical parts homing. Smart Clot’s optical system is composed of an objective (RMS20X;working distance: 2.1 mm, numerical aperture: 0.5, cover correction: 0.17 mm; Olympus Corporation, Tokyo, Japan), a green LED (Mic-LED-550A; peak λ: 544 nm, FWHM: 90 nm; Prizmatix, Holon, Israel), a blue LED (Mic-LED-500A; peak λ: 498 nm, FWHM: 29 nm; Prizmatix, Holon, Israel), an LED illuminator (LSQ-00-050-2-W-24V, TMS LITE, Sungai Ara Penang, Malaysia), two excitation filters (ET546/22x and ET480/30x, Chroma Technology, Bellows Falls, VT, USA) and one emission filter (59004m, Chroma Technology, Bellows Falls, VT, USA).

The Smart Clot software is able to manage the automated mechanical processes carried out by the instrument during the test. Furthermore, it is able to analyze the acquired images, providing quantitative outputs.

The Smart Clot Automation Kit includes 5 reagents (Table 1), a set of tips for the automated pipette, a waste container and a single-use cartridge made of medical-grade silicone and equipped with a 2.5 mL syringe and a glass slide (Figure 1B,C). The syringe’s piston is connected to a motor, which allows suction flow generation. The mounted glass slide allows the creation of, inside the cartridge perfusion chamber, a 21 mm long micro-channel with a rectangular section (0.2 × 2.1 mm H × W). The shear rate was calculated using the following formula:

$${\dot{\gamma }}_{w}=\frac{4Q}{\pi r^{3}}$$

where ${\dot{\gamma }}_{w}$ is the shear rate at the wall of the micro-channel, *Q* is the volumetric flow rate through the micro-channel and *r* is the radius of the micro-channel.

A research use only (RUO) development version of the Smart Clot system, as shown in Figure 1, was used for the preliminary experiments.

### 2.2. Optical System Calibration

InSpeck Microscope Image Intensity Calibration Kits (Thermo Fisher Scientific, Waltham, MA, USA) were used for the Smart Clot’s optical system calibration. InSpeck beads consist of polystyrene microspheres with different fluorescence intensities, which can be used as standards for the calibration of epifluorescence microscopes. 

Here, 2.5 µm diameter beads have been selected due to their size, comparable of that of platelets. The InSpeck Microscope Image Intensity Calibration Kits include seven vials of beads at different relative fluorescent intensities: 0, 0.3, 1, 3, 10, 30 and 100%. The 0% relative fluorescent intensity beads were used as a control. The 0.3, 1 and 3% relative fluorescent intensity beads (very low fluorescent intensity) were considered unnecessary for the Smart Clot fluorescence calibration, since their relative fluorescent intensities were very close to 0%. Given single adhered platelets’ fluorescent intensity, acquired by Smart Clot, around 5% (while the maximum mean fluorescent intensity expected is around 90%), for the purpose of the Smart Clot calibration, beads at 10, 30 and 100% relative fluorescent intensity were considered more relevant, in addition to preventing saturation at the end of the test.

**Figure 2:**
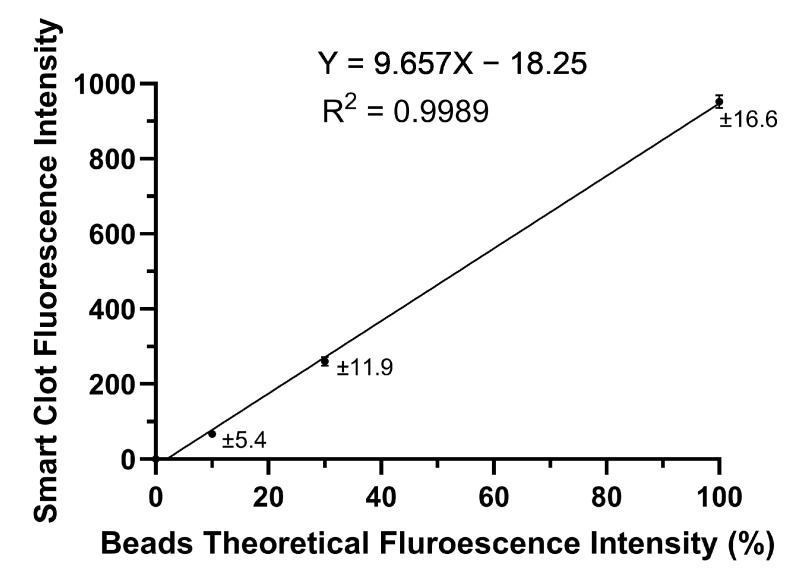
Relationship between Smart Clot’s fluorescent intensity values and the InSpeck Green beads at different fluorescent intensities. The Smart Clot fluorescence intensity is represented as the mean of six repeated measures for every point. Bars represent the SEM (standard error of the mean), which is also represented numerically for every point. The equation for the simple linear regression is shown above the graph.

Five μL of InSpeck Green beads (Ex peak 505 nm, Em peak 515 nm) at different intensities of fluorescence (0%, 10%, 30%, 100%) were centrally placed on glass slides assembled with a coverslip. The fluorescence intensity was then measured with the Smart Clot optical system, obtaining the output shown in Figure 2. The fluorescence intensity values calculated by Smart Clot show a linear relationship with the theoretical relative fluorescence of the beads at different intensities (R^2^ = 0.9989). The same experiment was repeated using InSpeck Orange beads (Ex peak 540 nm, Em peak 560 nm), which confirmed the linear relationship. To maintain standard acquisition conditions, a gain–exposure ratio of 1:4 was set.

### 2.3. Test Procedure

1. Sample and Reagent Loading

The blood sample is placed in the sample loading well. Reagents from the Smart Clot Automation Kit are placed in the reagent loading wells, following the sequence provided by the manufacturer. The disposable cartridge is placed in the cartridge container. The single-use waste container is placed in the waste container module.

2. Automated Test

For the following description, the Default Smart Clot Protocol is considered. Reagents A to F and sample are manually loaded in the reagents and sample support module of Smart Clot. From there, Smart Clot’s automated pipette handles all the following liquid handling steps. The reagents, once loaded, last for the daily activities. The first step in the test is the addition of 200 µL of Reagent C into the cartridge reservoir and its aspiration, in the flow chamber, at a shear rate of 500 s^−1^. An incubation of 7 min is then carried out for the purpose of cartridge heating to 37 °C and stabilization of the Reagent C coating on the cartridge glass slide bottom surface, which acts as the micro-channel’s roof. During incubation, Reagent P is added to the blood sample and the Camera Autofocus phase is carried out using physical marks present on the surface of the glass slide. After the incubation time, the Washing Solution is added to the cartridge reservoir and aspirated at a shear rate of 1500 s^−1^. Reagents A and F are then added to the cartridge reservoir, followed by the blood sample, thus composing the sample mixture. The cartridge is then automatically moved under the optical and recording system, while the sample mixture is aspirated at a shear rate of 300 s^−1^. The Camera Autofocus phase data are automatically adjusted, in the first step of the acquisition phase, in a fine focus phase carried out on the first-adhered platelets signal. The LEDs excite alternately the DiOC6(3) and Alexa Fluor 546 Conjugated Fibrinogen in the sample while the camera-equipped epifluorescent microscope receives the fluorescent signal emitted by the fluorochromes and records grayscale images of the flowing sample. The two fluorochromes used for the detection of platelets and fibrin(ogen) do not have overlapping emission spectra. Platelet adhesion/aggregation and fibrin(ogen) formation on the surface of the glass slide are visualized at the rate of 1 image/second, alternating between the two acquisition channels, corresponding to the different wavelengths of the fluorochromes used; thus, the time interval between two images in the platelet or fibrin(ogen) stacks is 1 s (a 1 s time interval between two images corresponds to a 2 s time resolution).

At the end of the 7 min acquisition phase, the system has collected a global set of images, half representing the platelets’ signal throughout the test and the other half representing the fibrin(ogen)’s signal throughout the test.

**Figure 3:**
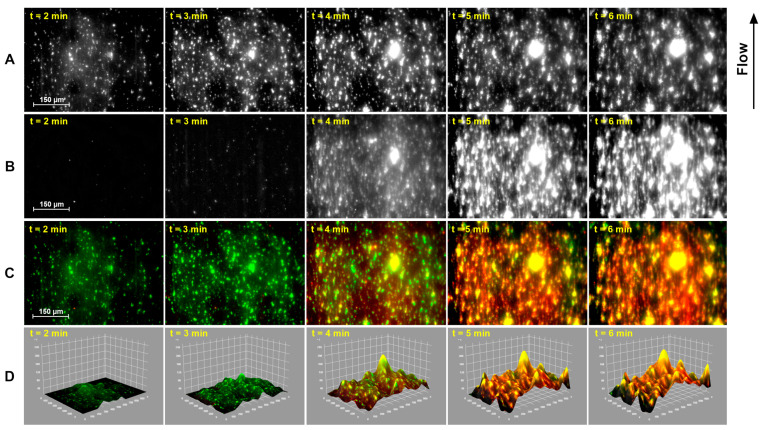
Example of images acquired from the Smart Clot system at different time points during a Smart Clot test: (**A**) platelet images; (**B**) fibrin(ogen) images; (**C**) platelet (green) and fibrin(ogen) (red) images merged and colored; and (**D**) pseudo-3D surface plot for each merged and colored image. A colored video of a full test can be found in the Supplementary Materials.

An example of the acquired images is shown in Figure 3, in which images acquired at the 2, 3, 4, 5 and 6 min time point for platelets and fibrin(ogen) are shown.

**Figure 4:**
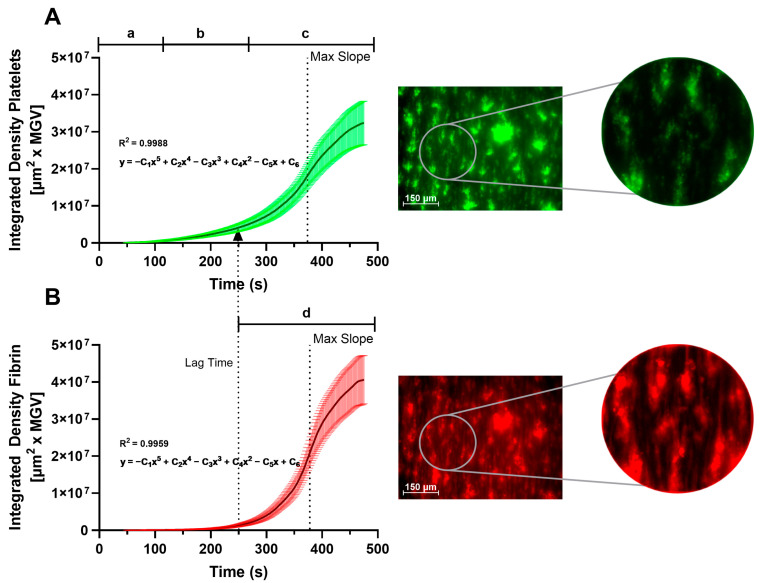
Integrated density curves obtained by averaging 43 Smart Clot tests on healthy donors for the different phases of the coagulation process (legend: a—platelets adhesion; b—platelets activation/aggregation; c—platelet thrombus formation; d—fibrin formation). Each curve is represented together with the 99% confidence interval for every point of the curve. The comparison of the zoomed-in images, shown on the right side of Panel (**A**) and Panel (**B**), highlights the presence, only in the fibrin(ogen) channel, of fibrils, which characterize fibrin. (**A**) Integrated density/time curve built from the platelet set of images. (**B**) Integrated density/time curve built from the fibrin(ogen) set of images. At T = Lag Time (beginning of the growth of the fibrin(ogen) curve), a dotted arrow is represented, indicating, in the upper graph (**A**), the moment of exponential growth of the platelet aggregate.

Colored merges and pseudo-3D plots, reported in Figure 3 and Figure 4, are obtained using ImageJ software v1.54i [17].

3. Image Processing

The images acquired during the test are analyzed by the Smart Clot software, which independently treats the platelets and fibrin(ogen) sets of images. During the first step, the images are binarized and a thresholding algorithm is used, which allows differentiation of the signal from the background. Each image in each set is analyzed two times, with different parameters, to obtain the optimal resolution for small and big objects. The resulting stacks are added together and the objects of dimension ≤2 pixels are removed. The last step consists of substituting the turned-on pixels with the original images’ grayscale.

### 2.4. Data Analysis and Output

In the Smart Clot System, each pixel equals 0.4 µm^2^, thus allowing the visualization of single adhered platelets (platelets’ mean diameter is 1.5–2.5 µm) [18]. The signal from the image’s sets is quantified and represented graphically. For each quantitative measurement obtained from the process of image analysis, one can construct a time course in which time 0 corresponds to the addition of calcium ions into the blood sample, promptly aspirated through the micro-analytical channel containing the cytoadhesive substance (Reagent C).

The parameters that Smart Clot calculates for each image, for both platelets and fibrin(ogen), are the pixel area (area of pixels turned on at a given time), mean gray value (mean gray value, on a scale of 0 to 255, of the turned-on pixel) and integrated density (obtained by multiplying the pixel area and mean gray value). Since the mean gray value (MGV) could be interpreted as the mean vertical size (relative to the coated glass slide) of the platelet aggregates or fibrin clots, the integrated density could be interpreted as an approximation of the 3-dimensional volume of the aggregate/clot (pseudo-3D).

### 2.5. Reagents

For the purpose of Reagent A preparation, calcium chloride lyophilized powder (Merck, Darmstadt, Germany) was resuspended in saline solution and used at a final concentration of 10 mM.

For the purpose of Reagent C preparation, acid-soluble type I collagen powder (Merck, Darmstadt, Germany) was suspended as described elsewhere [19] and its concentration was determined by Nanodrop measurement. The optimal concentration was considered to be 1–1.5 mg/mL.

For the purpose of Reagent F preparation, fibrinogen from human plasma, Alexa Fluor™ 546 Conjugate (Thermo Fisher Scientific, Waltham, MA, USA), was prepared as per the product manual. Subsequent dilutions were performed in DPBS. The fibrinogen–Alexa conjugate was used at a final concentration of 10 µg/mL.

For the purpose of Reagent P preparation, DiOC6(3) iodide powder (Thermo Fisher Scientific, Waltham, MA, USA) was resuspended as per the product manual [20]. Subsequent dilutions were performed in DMSO. DiOC6(3) was used at a final concentration of 0.87 µM. The DMSO volume was kept at <1% to prevent effects on the sample.

Indomethacin is a COX-1 and COX-2 inhibitor, which blocks platelet prostaglandins and Thromboxane A2 production, with an inhibitory effect on platelet activation and aggregation induced by collagen and ADP [21,22]. Indomethacin powder (Merck, Darmstadt, DEU) was resuspended in 100% ethanol and added to the blood samples, 5 min prior to the tests, at a final concentration of 36 µg/mL. The amount of Indomethacin solution added to the samples was less than 1% of the final volume. To the control blood sample was added the same amount of pure ethanol (with no Indomethacin).

Sold under the brand name Aggrastat^®^, Tirofiban is a specific platelet integrin α_2_b/β_3_ inhibitor (blocking fibrinogen and von Willebrand factor binding) with potent antiplatelet pharmacological activity in patients with acute cardiovascular diseases [22,23,24]. Aggrastat^®^ (Medicure, Winnipeg, MB, Canada) was added to the blood samples, prior to the tests, at a final concentration of 0.4 µg/mL.

Lepirudin, a recombinant hirudin, is a direct and highly specific thrombin inhibitor that simultaneously binds the active site and exosite 1 domain of thrombin [25]. Lepirudin (Bachem AG, Bubendorf, Switzerland) was diluted with salt solution and added to the blood samples, prior to the test, at final concentrations of 50, 200, 400 and 800 nM.

Unfractionated Heparin solution (Epsodilave, Pfizer, New York, NY, USA) was diluted with salt solution and added to the blood samples, 5 min prior to the tests, at final concentrations of 0.05, 0.5, 0.75, 1 and 2 IU/mL.

### 2.6. Blood Sampling

The subjects for this study were healthy volunteers, ranging from 18 to 65 years, with no ongoing pharmacological treatments in the last month and with no underlying conditions that could alter hemostasis, according to blood donors’ enrolment rules of Italy’s Health Minister (DLGS Nov 2015). All the donors signed a written informed consent form. This study was part of the project approved by the CEUR (Unique Regional Friulian Ethics Committee), under code CRO-2017-31. Blood samples were obtained from a peripheral vein of the arm into sampling tubes containing 3.2% sodium citrate anticoagulant. Per standard phlebotomy procedures, the first tube was always discarded. The remaining tubes were agitated briefly through inversion and maintained at room temperature (20–25 °C) until testing. The blood samples were analyzed within 2.5 h from the sampling.

### 2.7. Statistics

All the statistical analyses were performed using GraphPad Prism 10.2.2 for Windows (Dotmatics, Boston, MA, USA).

## 3. Results

### 3.1. Smart Clot’s Approach to Global Hemostasis Assessment

Figure 3 shows examples of images acquired from the Smart Clot system during a Smart Clot test. Figure 3A represents platelet images at time intervals of one minute (starting from minute 2 of the test). Figure 3B represents fibrin(ogen) images at the same time intervals. Figure 3C represents colored merges, for each time point, of the platelets(green) and fibrin (red) signal. Figure 3D represents, for each time point, the interactive pseudo-3D surface plots for each merged colored image. A video showing the merged and colored images of a complete test (different from the one showed in this image) can be found in the Supplementary Materials. The figure shows that fibrin starts to form only when the platelet aggregates reach a certain size, about 4 min into the test. This phenomenon is especially seen on the merged and colored images (Figure 3C) and in the full test video attached. The growing fibrin mesh tends to cover every zone of the image, even where platelet aggregates are not present. From the pseudo-3D plots (Figure 3D), it is possible to observe how fibrin mesh growth, favored by the turbulent sample flow, takes place mostly at the platelet aggregates’ base. The tallest aggregates show co-localization with a significant platelet component, favored by faster flow.

Figure 4 shows the integrated density curves obtained by averaging 43 Smart Clot tests on healthy donors. Fifth-order polynomial fits were built on the average curve obtained. The R^2^ value is 0.99 for both polynomial fits. For platelets, the polynomial equation is:

$$y=-0.001x^{5}+0.537x^{4}-83.06x^{3}+5544x^{2}-118881x+7.6^{5}$$

For fibrin(ogen), the polynomial equation is:

$$y=-0.002x^{5}+1.144x^{4}-180.47x^{3}+11666x^{2}-279124x+2^{6}$$

The integrated density is a parameter that takes into account, for each image, the amount of fluorescence and the area covered by fluorescence. The Max Slope (Table 2) and Lag Time (Table 2) are highlighted in both graphs. The Lag Time represents the moment right before fibrin(ogen) formation, which is dependent on endogenous thrombin generation on the surface of platelet aggregates (as seen in Figure 3). The arrow (corresponding to T = Lag Time) that connects the fibrin formation curve with the platelet aggregate growth curve represents the starting time of maximum platelet stimulation due to endogenous thrombin formation and the subsequent exponential growth of the aggregate, until it reaches a plateau [26,27].

From the platelet and fibrin(ogen) signal curves, the Smart Clot software automatically calculates different parameters related to the fluorescence intensity, area covered in fluorescence, reaction kinetics and dimension of fluorescent aggregates. The Maximum Velocity (MaxSlope) of the curves is automatically calculated by the Smart Clot software, providing information about the reactions’ kinetics. Only for the fibrin(ogen) curves, the Lag Time parameter is calculated, which represents the starting time of the formation of the fibrin clot. For both curves, the Smart Clot software also calculates the area under the curve of the output parameters, as well as their maximum and final value. Smart Clot’s outputs taken into consideration for this work and their physiological meaning are listed in Table 2.

### 3.2. Effect of Platelet Function Inhibitors on Primary Hemostasis and on Smart Clot’s Output

**Figure 5:**
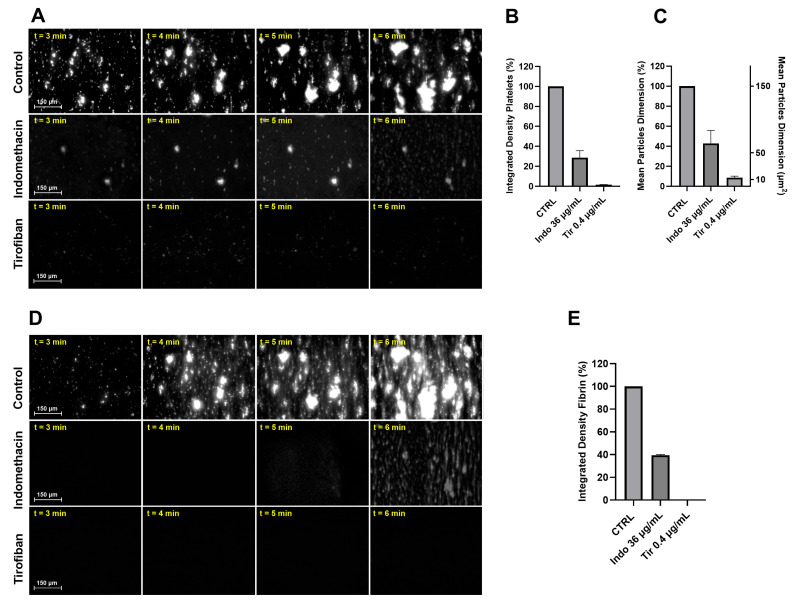
Effect of Indomethacin and Tirofiban inhibition on platelet aggregation and fibrin(ogen) formation. (**A**) Platelet channel fluorescent images taken from three different tests at different timings. (**B**) Comparison of Integrated Density Platelets of Indomethacin and Tirofiban treatments (2 tests each) vs. daily Control (100%). Bars represent SEM. (**C**) Comparison of Mean Particles Dimension of Indomethacin and Tirofiban treatments (2 tests each) vs. daily Control (100%). On the right y-axis, Mean Particles Dimension is expressed as µm^2^. Bars represent SEM. (**D**) Fibrin(ogen) channel fluorescent images taken from three different tests at different timings. (**E**) Comparison of Integrated Density Fibrin of Indomethacin and Tirofiban treatments (2 tests each) vs. daily Control (100%). Bars represented SEM. The inhibitory concentrations of Indomethacin and Tirofiban on platelet activation/aggregation have been chosen as suggested and confirmed by literature (see Section 2). Bars represent SEM.

The inhibition of platelet aggregation was tested with Indomethacin and Tirofiban. Indomethacin is a COX-1 and COX-2 inhibitor with antiplatelet pharmacological activity. Tirofiban, sold under the brand name Aggrastat^®^, is a glycoprotein α_2_b/β_3_ inhibitor (blocking fibrinogen and von Willebrand factor binding) with antiplatelet pharmacological activity. The purpose of these experiments was to evaluate the ability of the Smart Clot test to detect the effect of Indomethacin and Tirofiban on platelet aggregation and fibrin formation. Indomethacin was added to whole blood samples prior to the test at a final concentration of 36 µg/mL, while Tirofiban was added to whole blood samples prior to the test at a final concentration of 0.4 µg/mL. The Integrated Density Platelets and Integrated Density Fibrin parameters were selected to investigate the inhibitory effect of Indomethacin and Tirofiban on both platelets’ aggregation and fibrin(ogen)’s formation. The results are shown in Figure 5. In the Indomethacin-treated samples, Smart Clot detected a ~70% reduction in platelet aggregation and a ~60% reduction in fibrin(ogen) formation compared to the control. In the Tirofiban-treated samples, Smart Clot detected a complete inhibition of both platelet aggregation and fibrin(ogen) formation. The Mean Particles Dimension parameter was also taken into consideration. Smart Clot detected a mean reduction in the particles’ diameters in the presence of Indomethacin (~60%, ~50 µm^2^) and Tirofiban (~90%, ~10 µm^2^) compared to the control (~150 µm^2^) [28]. In the case of the Tirofiban treatment, only single adhering platelets were observed. Furthermore, the adhesion of these single platelets was highly unstable.

These findings confirm the ability of the Smart Clot system to detect Indomethacin’s and Tirofiban’s direct antiplatelet activities. Furthermore, Smart Clot demonstrates their inhibition of fibrin(ogen) formation as well. In fact, especially with the Tirofiban treatment, which completely inhibits platelet aggregation, Smart Clot detects a dramatic blockage of fibrin formation.

### 3.3. Effect of Thrombin Inhibitors on Secondary Hemostasis and on Smart Clot’s Output

**Figure 6:**
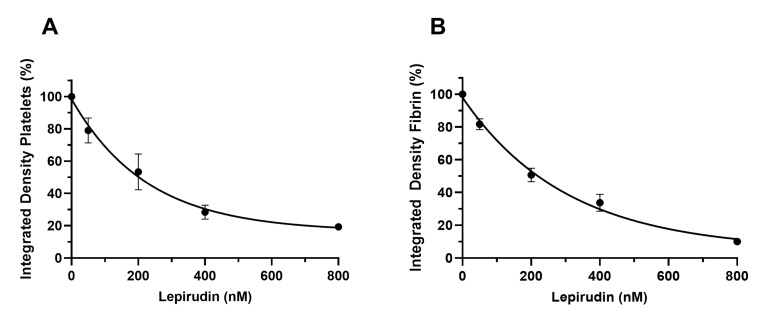
Dose–response curves of Lepirudin. The integrated density is expressed as % on sample with no treatment (set to 100%). Each integrated density value represents the mean of three repeated measures taken at defined time points during the test. Bars represent SEM. (**A**) Lepirudin’s dose-dependent effect on platelet thrombus formation. (**B**) Lepirudin’s dose-dependent effect on fibrin(ogen) formation.

Lepirudin-based thrombin inactivation was tested. Lepirudin is a direct and highly specific thrombin inhibitor. The purpose of these experiments was to evaluate the ability of the Smart Clot test to detect, in a dose-dependent way, the inhibition of platelet aggregation and fibrin(ogen) formation induced by Lepirudin. Lepirudin was added to whole blood samples prior to the test at concentrations 0, 50, 200, 400 and 800 nM. The Integrated Density parameter represents the total amount of platelet aggregation or fibrin(ogen) formation at the end of the test. The results are shown in Figure 6. The lowest Lepirudin concentration used (50 nM) led to a 20% decrease in both platelet aggregation and fibrin(ogen) formation. The highest Lepirudin concentration used (800 nM) led to an 80% decrease in platelet aggregation and a 90% decrease in fibrin(ogen) formation. The direct and specific inhibition of thrombin by Lepirudin led to a dose-dependent decrease in both platelet aggregation and fibrin(ogen) formation. The Smart Clot test was able to detect the dose-dependent inhibition of thrombin by Lepirudin, proving to be highly sensitive to endogenous thrombin formation.

**Figure 7:**
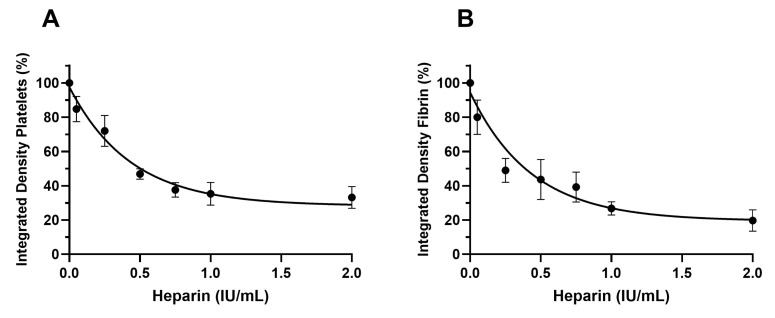
Dose–response curves of Heparin. The integrated density is expressed as % on sample with no treatment (set to 100%). Each integrated density value represents the mean of at least three replicates. Bars represent SEM. (**A**) Heparin’s dose-dependent effect on platelets aggregation. (**B**) Heparin’s dose-dependent effect on fibrin(ogen) formation.

In a similar setting, thrombin inactivation by unfractionated Heparin was tested. The purpose of these experiments was to evaluate the ability of the Smart Clot test to detect, in a dose-dependent way, the anticoagulant effect of Heparin. Unfractionated Heparin was added to whole blood samples prior to the test at concentrations 0, 0.05, 0.5, 0.75, 1 and 2 IU/mL (this range represents a real-world model of anticoagulation for many clinical procedures). The Integrated Density parameter represents the total amount of platelet aggregation or fibrin(ogen) formation at the end of the test. The results are shown in Figure 7. The lowest Heparin concentration used (0.05 IU/mL) led to a 15% decrease in platelet aggregation and to a 20% decrease in fibrin(ogen) formation. The highest Heparin concentration used (2 IU/mL) led to a 70% decrease in platelet aggregation and an 80% decrease in fibrin(ogen) formation. Heparin’s anticoagulant effect leads to a dose-dependent decrease in both platelet aggregation and fibrin(ogen) formation. The Smart Clot test was able to detect the dose-dependent anticoagulant effect of Heparin, confirming, together with the results obtained with Lepirudin, it to be highly sensitive to endogenous thrombin formation.

## 4. Discussion

Although widely used, the viscoelastic tests available on the market suffer from some limitations. In all the existing devices, clot formation is triggered by specific activators and translated into physical properties by the movement of a detector pin (oscillating or rotating) or by other physical techniques. Without the application of specific activators (native tests), the process of clot formation would require a very long time (about 1 h to be completed). Actually, these devices try to simulate the dynamic process of clot formation by triggering a thrombin burst using kaolin or tissue factor. This will activate platelets through their protease activating receptors (PARs) and platelets will produce additional thrombin on their surface and express activated receptors. Thrombin, in turn, will activate fibrinogen to fibrin, producing a fibrin network stabilized by factor XIII. Finally, the clot is constituted by the fibrin network and the platelet aggregation. However, this simulation of the natural process of clot formation is a sort of approximation. In the real process, the collagen and tissue factor exposed in a damaged endothelium will produce a preliminary platelet activation and formation of small quantities of thrombin. This small amount of thrombin will activate the platelet PARs, giving rise to the following steps of clot formation. Therefore, the simulated model of viscoelastic tests jumps over the preliminary step of platelet activation as the main player in thrombin generation.

Additionally, they do not take into account the flow-dependent platelet activation linked to the shear rate (SR). Platelet adhesion and activation are strongly dependent on the SR [29,30,31,32], and thrombin generation is increased under conditions of a high SR [33]. The existing viscoelastic tests work at a very low SR (0.5 s^−1^) that cannot be found in any part of the natural circulation. For this reason, they invariably require some sort of chemical activation.

The Smart Clot system is based on a totally different approach with the aim of reproducing, as closely as possible, what happens in the human body: clot formation is triggered by (a) collagen, mimicking the exposure of damaged endothelium and by (b) an SR of 300 s^−1^.Previous studies have already demonstrated that an SR of 80 s^−1^ is sufficient to trigger clot formation without chemical activation after 1 min only [34]. An SR of 300 s^−1^ corresponds to the value found at the level of venous circulation in physiological conditions.

Collagens mediate both platelet adhesion and activation through direct and indirect mechanisms influenced by fluid dynamic conditions. Under the Smart Clot shear rate conditions (300 s^−1^), the initial interaction between the circulating platelets and the vessel wall is mediated by the binding of glycoprotein (GP) Ib to von Willebrand factor (VWF) immobilized onto collagen fibrils [35,36]. The GPIb-VWF interaction promotes the initial tethering, but subsequent firm platelet adhesion is also supported by two platelet collagen receptors, glycoprotein VI (GPVI) and the integrin α2β1 [37].

Our preliminary data generate the hypothesis that the Smart Clot model of activator-free blood activation should represent the above described physiological dynamic pattern of clot formation. Actually, the different chemical products used during a Smart Clot test, not being exogenous direct activators, cannot be considered powerful coagulation activators: collagen is stratified on the cartridge only to simulate a damaged endothelium condition; reagent A is calcium chloride, which is the standard way to reverse the anticoagulant effects of citrate; and reagents P and F are fluorescent labelers of platelets and fibrinogen, respectively.

The possibility of detecting the separate contribution of platelets and fibrin to clot formation in a single test is a potential strength of the Smart Clot system. The commercially available viscoelastic tests require more than one test to do this and platelet function analyzers are often used in addition to viscoelastic tests in order to complete the blood clot analysis [1]. Additionally, the existing viscoelastic tests require a platelet inhibitor for the fibrinogen contribution assessment [38] and the degree of inhibition varies from device to device [39]. Some insight into platelets’ contribution to clot strength using viscoelastic tests is indirectly based on the difference between the maximum clot strength and the fibrinogen component. This measure requires kaolin, a powerful thrombin activator, which in turns triggers platelet aggregation regardless of drug-induced platelet dysfunction [40]. Additionally, there is no consensus on how to assess the difference between the maximum clot strength and fibrinogen’s contribution: the simple difference in millimeters or the difference in the shear modulus (hPa) [41].

In this work, we tested the ability of the Smart Clot system to evaluate the in vitro effect of the antiplatelet drugs Indomethacin and Tirofiban and antithrombin drugs Lepirudin and Heparin. Antiplatelet drugs have a direct effect on primary hemostasis (formation of platelet plug), while antithrombin drugs have a direct effect on secondary hemostasis (fibrin mesh formation). Primary and secondary hemostasis mutually influence each other, triggering stronger effects than the two processes separately [27]. The Smart Clot system was capable of detecting this interplay between platelets and coagulation.

As mentioned in the Discussion, there are other devices, still not commercially available, that avoid the use of coagulation activators and are based on microfluidic analyses and shear-stress-based activation, so this principle is not a novelty of Smart Clot. However, considerable differences exist among the various devices, especially regarding the output of the machine. A common feature of these (and the Smart Clot) devices is that they produce a “reaction time” that is the time from the beginning of the test to the first fibrin network formation. Different authors have tested the reaction time under pharmacological inhibition of the coagulation (antiplatelet agents, warfarin, Heparin) and found results similar to ours. The main difference relies on the measure of the platelet/fibrin contribution to the final clot composition. This is automatically visualized in the Smart Clot system through labeling of both platelets and fibrin, and translated into a measure of the relative contribution. Conversely, the other devices simply measured the reaction time in warfarin-treated patients [8] or under Heparin’s effects [9] without addressing the subsequent clot strength. A more comprehensive device [7] has the potential for visualization of fibrin’s and platelets’ contribution through the application of microscopy; however, as the authors stated, this an option, and the results of the different tests produced by the authors are limited to the reaction times.

It is not the purpose of the present study to claim the superiority of the Smart Clot system with respect to the other commercially or not commercially available devices, but to present a technology that certainly is different from the existing ones. A strength of our study is having performed and presented ex vivo human data in a large series of healthy subjects, and specific in vitro tests demonstrating that the Smart Clot-derived parameters are sensitive to both antiplatelet drugs and thrombin/fibrin-inactivating drugs. The major limitation is that we did not compare our results to those obtained with the standard POC tests available. An ongoing study from our group is addressing patients under dual antiplatelet therapy by measuring platelet inhibition with Smart Clot vs. Multiplate, and the results will be available soon. Other comparative tests will include (i) ex vivo analysis of the effects of thrombin-inhibiting drugs (unfractionated and low molecular weight heparin; warfarin; direct oral anticoagulants); (ii) ex vivo analysis of the patterns of thrombocytopenia, hypofibrinogenemia, and the effects of the correction of both the conditions; and (iii) ex vivo analysis of the effects of exposure to clinical settings where a number of factors induce an acquired coagulopathy (cardiac surgery with cardiopulmonary bypass; liver transplant; severe sepsis; and others).

These, and other experiments involving comparisons with conventional point-of-care coagulation devices, will be the focus of the next steps in the validation of the Smart Clot system.

## Figures and Tables

**Table 1:** Smart Clot Automation Kit–Platelets and Fibrin Detection reagents.

Reagent Name	Reagent Composition	Reagent Scope
Reagent A	Calcium chloride	Re-calcification of the sample
Reagent C	Acid-soluble type I collagen	Coating of the glass slide
Reagent F	Fibrinogen from human plasma, Alexa Fluor 546 conjugate	Fluorescent labeling of fibrin(ogen)
Reagent P	DiOC6(3) solution	Fluorescent labeling of platelets
Washing Solution	Saline solution 0.9%	Washing of the perfusion chamber

**Table 2:** List of Smart Clot’s outputs, as calculated by the Smart Clot software.

Smart Clot Output	Parameter Description	Units	Physiological Meaning
ID PLTS	Integrated Density Platelets	µm^2^ × MGV (mean gray value)	Pseudo-3D size of platelet thrombus
ID FIB	Integrated Density Fibrin	µm^2^ × MGV	Pseudo-3D size of fibrin clot
Dim Part	Mean Dimension of Particles (only Platelets)	µm^2^	Platelets’ aggregation potential of the patient
Lag Time	Lag Time (only Fibrin) (3-fold SD growth compared to baseline)	Time (s)	Starting time of fibrin formation
Max Slope	Maximum Velocity (curve’s tangent maximum value)	$\frac{{µm}^{2}\times MGV}{s}$	Kinetics of the coagulation process

## Data Availability

The raw data supporting the conclusions of this article will be made available by the authors on request.

## Supplementary Materials

The following supporting information can be downloaded at https://www.mdpi.com/article/10.3390/bios14110518/s1, Video S1: Video of a full test acquired from the Smart Clot system during a Smart Clot test.
